# Genomic insights into the re-emergence of chikungunya virus on Réunion Island, France, 2024 to 2025

**DOI:** 10.2807/1560-7917.ES.2025.30.22.2500344

**Published:** 2025-06-05

**Authors:** Etienne Frumence, Géraldine Piorkowski, Nicolas Traversier, Rayane Amaral, Muriel Vincent, Ambroise Mercier, Nazli Ayhan, Laurent Souply, Laura Pezzi, Clément Lier, Gilda Grard, Guillaume André Durand, Chikungunya genomics diagnostic laboratory network, Xavier Deparis, Fabian Thouillot, Xavier de Lamballerie, Raphaelle Klitting, Marie-Christine Jaffar-Bandjee, Chikungunya genomics diagnostic laboratory network, Laura Verdurme, Paul-Emile Gin, Mahery Ramiandrisoa, Paul Séraphin, Audrey Pignolet, Thierry Benoit Cattin, Yann Pepino

**Affiliations:** 1 Associated National Reference Center for Arboviruses, CHU-Réunion, Saint-Denis, Réunion, France; 2 Laboratoire de microbiologie, CHU de la Réunion-Site Nord, Saint-Denis, Réunion, France; 3 Unité des Virus Émergents (UVE: Aix-Marseille Univ, Università di Corsica, IRD 190, Inserm 1207, IRBA), France; 4 National Reference Center for Arboviruses, Inserm-IRBA, Marseille, France; 5 Santé Publique France, Saint Denis, Réunion, France; 6 Members of the Chikungunya genomics diagnostic laboratory network are listed under Collaborators and at the end of the article; 7 Agence Régionale de Santé (ARS) de La Réunion, Saint-Denis, Réunion, France

**Keywords:** arbovirus, Chikungunya, surveillance, emergence, molecular epidemiology, Genomic analysis

## Abstract

Chikungunya virus re-emerged on Réunion Island in August 2024, 18 years after a first major outbreak. Analysis of 173 genomes from the current epidemic reveals a monophyletic clade with mutations linked to adaptation to *Aedes albopictus* mosquitoes, including E1-A226V. Bayesian inference suggests only brief cryptic circulation before detection. The same lineage was also detected on Mayotte Island in March 2025. Continued spread and confirmed travel-related cases in mainland France and globally highlight the risk of wider regional and international dissemination.

Chikungunya virus (CHIKV) is an arbovirus transmitted primarily by *Aedes (Ae.) aegypti* and *Ae. albopictus* mosquitoes. In 2005–06, Réunion Island was impacted by a major chikungunya epidemic [1,2], but since then, very few cases of chikungunya have been recorded, with no evidence of autochthonous transmission between 2011 and 2024 [3]. In August 2024, chikungunya virus (CHIKV) was detected again on Réunion Island. Here, we provide a brief description of the epidemic, and describe the virus lineage responsible for the current epidemic using genomic data.

## Chikungunya outbreak progression on Réunion Island 

**Figure 1:**
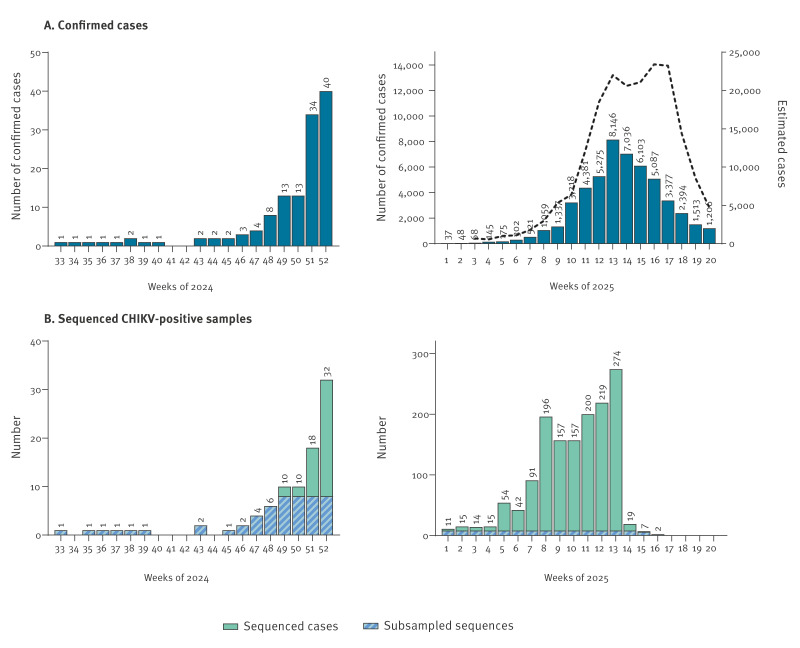
Temporal distribution of confirmed chikungunya cases (n = 51,558) and sequenced CHIKV genomes (n = 1,564), Réunion Island, France, August 2024–May 2025

The first confirmed chikungunya case , i.e. laboratory-confirmed by RT-PCR, was identified on 23 August in Saint-Paul on the west side of the island during the austral winter [4,5]. Circulation of CHIKV remained low (< 4 chikungunya cases/week) in the west and south-west of the island until the more humid summer season in late 2024. By December, transmission intensified (up to 40 cases/week) and expanded across most regions of the island (Figure 1A, left panel). Up to 18 May 2025, more than 51,000 confirmed cases and over 188,600 suspected cases — defined as clinically compatible cases presenting with fever ≥ 38.5 °C, with or without headache, joint pain and rash, in the absence of another identified infectious cause — have been reported among a population estimated at around 896,000 inhabitants [6]. The epidemic peaked between weeks 13 and 17 of 2025, with up to > 23,000 estimated cases/week (Figure 1A, right panel). 

CHIKV: chikungunya virus.

A. Weekly number of confirmed chikungunya cases based on sampling date in 2024 and 2025. Estimated numbers of chikungunya-related consultations in private medical practices for 2025 are shown as a dotted line. These estimates are based on weekly reports from a network of sentinel physicians who record all clinically compatible cases, defined as fever ≥ 38.5 °C, with or without headache, joint pain and rash, in the absence of another identified infectious cause. Data were provided by the regional health agency (Agence Régionale de Santé, ARS Réunion) and processed by the French national public health agency (Santé publique France Réunion, SPF).

B. Weekly number of CHIKV-positive samples sequenced from Réunion Island, based on sampling date, in 2024 and 2025, along with the proportion of sequences included in the representative subsample of 173 sequences from Réunion Island.

## Identification of the CHIKV lineage on Réunion Island and its detection on Mayotte

After more than 10 years with no autochthonous virus circulation, it is likely that the new emergence of CHIKV on Réunion Island is associated with one -or multiple- recent virus introduction(s) into the island. To identify the virus lineage(s) associated with the current epidemic, we sequenced all laboratory-confirmed samples received by the French National Reference Centre (NRC) for arboviruses in Réunion Island from both private laboratories and public hospitals on Réunion Island (n = 1,564 genomes by 5 May 2025). We analysed a subset of 173 virus genomes randomly sampled (up to 8/week) between August 2024 and April 2025 (Figure 1B). Sample metadata are provided in Supplementary Table S2. We also included 16 sequences from travellers returning from Réunion Island to mainland France (analysed by the NRC in mainland France) which were selected by convenience sampling. Sample metadata are provided in Supplementary Table S4. 

**Figure 2:**
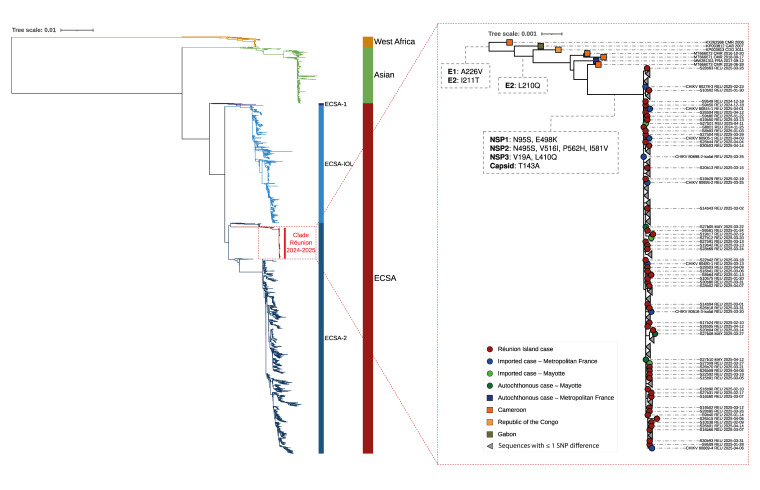
Phylogenetic tree of CHIKV genomes from 1952–2025 (n = 2,729) and the Réunion Island clade, France, 2024–2025 (n = 201)

CHIKV: chikungunya virus; ECSA: East-Central-South African genotype; NSP: non-structural protein.

By inferring the phylogenetic relationships between virus genomes from Réunion Island and a dataset encompassing CHIKV global diversity, we found that the sequences all grouped in a monophyletic clade belonging to the East-Central-South African (ECSA) genotype and the ECSA-2 lineage (Figure 2) [7]. This clade was distinct from the one that circulated during the epidemic of 2005–06 on Réunion Island, which belonged to the ECSA-IOL (Indian Ocean lineage), and had, as its closest phylogenetic relatives, sequences from the Central African region, including strains from Cameroon 2016–18, as well as a strain from the 2017 autochthonous chikungunya outbreak in mainland France [8].

Maximum likelihood phylogenetic reconstruction was performed using IQ-TREE with the best-fitting nucleotide substitution model and 1,000 ultrafast bootstrap replicates. The tree includes 2,528 CHIKV sequences retrieved from GenBank on 27 March 2025, each covering more than 85% of the open reading frame. The tree was rooted on the West African genotype. A focus is presented on the 2024–25 Réunion clade, and on the closest sequences harbouring the E1-A226V mutation. Identical or near-identical sequences (≤ 1 SNP difference; average branch length ≤ 0.0001) were collapsed into grey triangles on the tree for clarity. Sequences are labelled according to their geographic origin. Major genotypes and lineages are indicated.

When examining the 189 CHIKV genomes from the current epidemic, we found that they all exhibited the E1-A226V mutation, which is known to increase transmission efficiency in Ae. albopictus mosquitoes [9]. This mutation was also present in the closest phylogenetic relatives within the ECSA-2 lineage (Figure 2) and likely emerged decades before the current outbreak, with the earliest sequence carrying this mutation dating back to 2006 (GenBank accession: KX262996, Cameroon, 2006). Additionally, we identified two other mutations linked to increased fitness in *Ae. albopictus*: E2-I211T, which provides a more favourable background for E1-A226V, and E2-L210Q, associated with enhanced viral dissemination in *Ae. albopictus* [10]. Both mutations were shared with the closest phylogenetic ancestors of the epidemic clade. Furthermore, 37 mutations specific to the Réunion lineage were detected, including nine non-synonymous mutations (eight in the non-structural proteins and one in the capsid) (Figure 2). Given the predominance of *Ae. albopictus* on Réunion Island, the presence in the strain that emerged on the island of mutations conferring a heightened ability to propagate in the main mosquito vector — E1-A226V in combination with E2-I211T and E2-L210Q — is likely to facilitate the local transmission of the virus and contribute to the scale and intensity of the current epidemic. 

By May 2025, CHIKV had also been detected locally on the islands of Mayotte and Mauritius [11]. To confirm these epidemiological observations, we generated three CHIKV sequences from cases acquired in Mayotte, and nine from travellers returning from Réunion Island to Mayotte and included them in our global phylogeny analysis (detailed information in Supplementary Table S4). As expected, all traveller sequences from Réunion clustered within the 2024–25 clade, as did sequences from local Mayotte cases (Figure 2), confirming that the epidemic lineage had now become established in Mayotte, likely following an introduction from an infected traveller.

## Limited undetected transmission of CHIKV on Réunion Island, 2024

The index case on Réunion Island was diagnosed on 23 August 2024, based on a sample collected on 14 August. The patient had no travel history or known link to any symptomatic or suspected case, implying a phase of undetected local circulation before diagnosis [4,5]. Previous viral emergence events have highlighted that local virus circulation may start long before the first cases are confirmed, leading to a delay between the occurrence of the primary case and the detection of the index case, also called ‘surveillance gap’, as observed during the emergence of Zika virus (ZIKV) in the Americas in 2015 [12]. Given the active surveillance of CHIKV on Réunion Island, which is conducted year-round [13], and the high proportion of symptomatic infections compared with what is typically observed with ZIKV infections, we expect this gap to be minimal for the current epidemic. 

To estimate the date of re-emergence of CHIKV on Réunion Island, we controlled for the presence of a molecular clock signal and selected a final subset of 187 CHIKV genomes from the current epidemic to evaluate the time of emergence of the epidemic clade with Bayesian inference. Details on the method used are provided in Supplementary Material S1. Using the best fitted model (Supplementary Table S3 and Supplementary Figure S7), we evaluated a mean evolutionary rate of 3.6*10^−4^ substitution/site/year (95% highest posterior density (HPD) interval: 2.6*10 ^− 4^–4.6*10^−4^), which is consistent with previous analyses of CHIKV [7,14], and estimated the time to the most recent ancestor of the epidemic clade to be around 7 August 2024 (95% HPD: 9 Jul 2024–14 Aug 2024). This result confirms our hypothesis of limited cryptic transmission before detection of the index case and, notably, coincides with the austral winter, when mosquito populations are low. 

## Discussion

Between October 2005 and December 2006, Réunion Island was affected by a major chikungunya epidemic estimated to have caused ca 266,000 cases (ca 34% of the population) and several thousand cases in other Indian Ocean islands [1,2]. Chikungunya virus circulation in 2005–06 extended beyond Réunion Island to neighbouring islands (Seychelles, Comoros, Mauritius, Mayotte, Madagascar), and to other continents, including Europe (Italy) and Asia (India), where it spread for over 2 years, causing an estimated 1.4 million cases [15,16]. After 18 years, without any local circulation, CHIKV is causing a new epidemic on Réunion Island. 

Our analysis of virus genomic data from the first phase of the current epidemic shows that it is driven by a unique virus lineage that likely circulated since August 2024. The successful local establishment of a CHIKV lineage during the austral winter on Réunion Island highlights the importance of continuous surveillance even in a period of low vector activity to enable timely public health responses. This strain has now spread to Mayotte and Mauritius. Although case trends appear to be decreasing, there is a sizeable risk of further spread to other Indian Ocean islands (Seychelles, Comoros, Madagascar) and a non-negligible risk of spread beyond via infected travellers. 

Previous analysis of the virus lineages that circulated in Indian Ocean islands in 2005–06 identified one dominant lineage belonging to the ECSA genotype, in which the E1-A226V mutation emerged during the epidemic, reaching a frequency of more than 90% among sequenced strains from Réunion Island by the end of 2006 [17]. As suggested by its rapid progression to dominance in the viral population, the E1-A226V mutation appeared to be an adaptation to *Ae. albopictus* mosquitoes, conferring increased midgut infectivity, enhanced dissemination to the salivary glands, and improved transmission efficiency [9]. The repeated emergence of E1-A226V in other regions and CHIKV lineages further suggests that this mutation provides a selective advantage for transmission in *Ae. albopictus* and may thus have contributed to the magnitude of the 2005–06 outbreak on Réunion Island. 

Here, we show that the virus lineage responsible for the current epidemic on the island also exhibits E1-A226V along with other mutations that likely facilitate propagation in *Ae. Albopictus*, E2-I211T and E2-L210Q. Since January 2025 and up to the end of May, more than 900 chikungunya cases had been reported in mainland France, most originating from Réunion Island [18]. Given the epidemic lineage’s ability to efficiently circulate in *Ae. albopictus* populations, and as this vector is present across a large part of Southern Europe with an expected increase in activity as the summer approaches, caution and sustained vigilance are warranted due to the potentially higher epidemic potential of this lineage.

## Conclusion

The CHIKV outbreak on Réunion Island underscores the critical need for sustained systematic testing alongside genomic surveillance. This dual approach is indispensable not only for monitoring CHIKV circulation but also for detecting potential co-circulation of other CHIKV strains or arboviruses, including dengue virus. Together, these strategies are essential to support timely and effective public health responses throughout Réunion Island and the broader Indian Ocean region.

## Data Availability

All virus sequences are accessible on Genbank (accession numbers specified in Supplementary Materials) and on Github (https://github.com/rklitting/CHIKV_Reunion_2025_RC). The xml and tree files used in this study are available on Github (same repository as above).

## Chikungunya genomics diagnostic laboratory network

Laura Verdurme (Laboratoire Cerba, Frépillon, France); Paul-Emile Gin (Inovie Laboratory, Réunion); Mahery Ramiandrisoa (Cerballiance, Réunion); Paul Séraphin (Bioaustral Laboratory, Réunion); Audrey Pignolet (Medical biology laboratory of Saint-Benoît, Réunion); Thierry Benoit Cattin (Centre Hospitalier de Mayotte); Yann Pepino (Centre Hospitalier, Ouest Réunion).

## Supplementary Data

412000 Supplement
