# Supplementary material for: Genomic insights into the re-emergence of chikungunya virus on Réunion Island, France, 2024 to 2025
Source: Euro Surveill. 2025 Jun 5;30(22):2500344. doi: 10.2807/1560-7917.ES.2025.30.22.2500344 (PMC12143119; doi:10.2807/1560-7917.ES.2025.30.22.2500344)
Supplement: Supplement [file 25-00344_FRUMENCE_Supplement.pdf]

Supplementary data:

This supplementary material is hosted by Eurosurveillance as supporting information alongside the article "Genomic Insights into the Re-emergence of CHIKV in Réunion Island (2024–2025)", on behalf of the authors, who remain responsible for the accuracy and appropriateness of the content. The same standards for ethics, copyright, attributions and permissions as for the article apply. Supplements are not edited by Eurosurveillance and the journal is not responsible for the maintenance of any links or email addresses provided therein.

Supplementary Material S1:

Sample selection

Arbovirus NRC – Réunion

Between 2024 and 2025, all CHIKV-positive samples tested at the associated Arbovirus French National Reference Center (NRC) in Réunion were sequenced as part of the arboviral genomic surveillance program implemented by the center since 2022, without any Ct value threshold criteria. From August 2024 to mid-February 2025, all CHIKV-positive cases detected on the island by private and public laboratories (see the Chikungunya Genomics Diagnostic Laboratory Network and Working Group) were systematically forwarded to the NRC for sequencing, whenever available. After mid-February, due to the sharp increase in case numbers, the various laboratory groups submitted a randomized selection of 10 to 30 positive samples per week. Meanwhile, all CHIKV-positive samples collected at Réunion Island University Hospital were automatically included for further analysis at the NRC. Additionally, a selection of positive cases detected on the nearby French island of Mayotte, including patients returning from travel to Réunion as well as autochthonous cases, was also sent for complementary analysis.

Arbovirus NRC – Mainland France

In 2024-2025, all Chikungunya positive samples tested at the French National Reference Center in mainland France (Marseille) with a Ct value below 33 with a possible infection origin in La Réunion island were sequenced as part of the arboviral genomic surveillance system implemented by the center since 2022.

Sequencing

Arbovirus NRC – Réunion

Complete CHIKV coding sequences were obtained using an in-house amplicon-based protocol on a MinION device (Oxford Nanopore Technologies, ONT). Briefly, viral RNA was extracted from serum and plasma samples using the NucliSENS® easyMAG kit on the eMAG automated system (bioMérieux). Reverse transcription was then performed with the LunaScript RT SuperMix kit (New England Biolabs, NEB). Multiplex PCRs were conducted in two separate pools using the Q5 Hot Start High-Fidelity 2X Master Mix (NEB), with a custom CHIKV primer scheme designed using the PrimalScheme tool (https://primalscheme.com) detailed in Supplementary Table S6. PCR conditions included an initial denaturation at 98 °C for 30 s, followed by 40 cycles of denaturation at 98 °C for 15 s, hybridization at 62 °C for 40 s with a slow temperature ramp (0.1 °C/s) down to 58 °C, a second hybridization step at 58 °C for 40 s, and elongation at 72 °C for 2 min. A final extension step was performed at 72 °C for 3 min. Amplicons were then pooled, quantified using the Quant-iT™ DNA Assay Kit (Thermo Fisher Scientific), and normalized to 10 ng/µL prior to barcoding. Barcoding was performed using the Rapid Barcoding Kit 96 V14 (SQK-RBK114.96, ONT) according to the manufacturer's protocol. Libraries were loaded onto R10.4.1 flow cells and sequenced on the ONT MinION platform. Raw sequencing data were basecalled using the latest super-accurate model, filtered for high-quality reads (mean Q-score >15), and demultiplexed using the MinKNOW software (ONT). Consensus genome sequences were generated using the ARTIC network's field bioinformatics pipeline (v1.6.1; https://github.com/artic-network/fieldbioinformatics), with the CHIKV reference genome NC_004162 (GenBank) as reference. Regions with insufficient coverage were masked with N characters. Only sequences with a complete coding sequence (CDS) and a minimum coverage depth of 100X were retained.

Arbovirus NRC – Mainland France

A specific set of primers (supplementary table S5) was used to generate eight overlapping amplicons spanning the entire CHIKV genome with the Superscript IV one step RT-PCR System (ThermoFisher Scientific). PCR mixes (final volume 25µL) contained 3µL of nucleic acid extract, 1,25µL of each primer (10µM), 12.5 µL of 2X Platinum SuperFi RT-PCR Master Mix, 6.5µL of RNAse free water and 0.5µL of SuperScript IV RT Mix. Amplifications were performed using the following conditions: 10 min at 55°C, 2 min at 98°C, followed by 40 cycles with the three following steps: 10 sec at 98°C, 10 sec at 55°C and 1.45 min at 68°C, and a final step at 68°C for 5 min.

The size of PCR products was controlled by gel electrophoresis. For each sample, an equimolar pool of all amplicons was prepared and purified using Monarch PCR & DNA Cleanup Kit (New England Biolabs). After Qubit quantification using the Qubit® dsDNA HS Assay Kit and Qubit 2.0 fluorometer (Thermo Fisher) amplicons were sonicated (Bioruptor®,

Diagenode, Liège, Belgium) into 250pb long fragments. Fragmented DNA was used for library building using the Ion Plus Fragment Library Kit with the AB Library Builder System (Thermo Fisher). To ensure the equimolar pooling of the barcoded samples, a real time PCR quantification step was performed using Ion Library TaqMan™ Quantitation Kit (Thermo Fisher). An emulsion PCR of the pools was performed, followed by loading on 530 chips using the automated Ion Chef instrument (Thermo Fisher), and sequencing using the S5 Ion torrent technology (Thermo Fisher), following manufacturer's instructions.

Read data were analyzed with an in-house Snakemake pipeline [1]. Read alignment was achieved using BWA MEM (v0.7.17, [2]) using, as a reference, a sequence from the current epidemic (Genbank accession: PV035814). Consensus sequences were called using the ivar (v1.3.1, [3]) consensus command, and a minimum coverage depth of 50x. Regions with insufficient coverage were masked with N characters.

Sequence datasets

To obtain a representative subset of sequences from the beginning of the outbreak in Réunion to its peak, samples collected between August 2024 and early April 2025 were subsampled. When possible, eight sequences per week were randomly selected using the augur filter tool included in the Nextstrain CLI. The resulting dataset of 173 sequences from Réunion Island (detailed in Supplementary Table S2) consists primarily of sequences obtained from community cases detected by private laboratories between August 2024 and January 2025, and predominantly from cases identified at the University Hospital of Réunion Island (CHU Réunion) from February 2025 onward.

All publicly available sequences for chikungunya virus were downloaded from the NCBI Nucleotide database, Genbank (keywords: "chikungunya virus"; database accessed on March 27th, 2025). We filtered the data by: (i) excluding sequences from laboratory strains (adapted, passaged multiple times, obtained from antiviral or vaccine experiments), (ii) excluding sequences that did not belong to the chikungunya virus species, (iii) keeping only sequences covering more than 85% of the open reading frame (ORF). The remaining 2 528 sequences were trimmed to their ORF, aligned using MAFFT (version 7.511, [4]) and inspected manually using the program AliView (version 1.0, [5]).

We combined this set –accounting for CHIKV global phylogenetic diversity– with 173 sequences from La Réunion, 16 sequences from cases imported into metropolitan France from La Réunion, and 12 sequences from Mayotte either imported from La Réunion (9) or from local cases (3) (detailed in Supplementary Table S4). We also built a smaller dataset for bayesian inference, including only sequences from Réunion island (local cases and cases imported to mainland France), totalling 189 sequences.

Phylogenetic analysis

For both alignments, we performed a Maximum-likelihood (ML) phylogenetic reconstruction with IQ-Tree (version 1.6.12, [7-8]), using the best-fit model identified by ModelFinder and assessed branch support using an ultrafast bootstrap approximation (UFBoot2) (1 000 replicates). The alignment (.fasta) and tree (.nexus) files for this analysis are available at https://github.com/rklitting/CHIKV_Reunion_2025_RC. Phylogenetic trees were annotated and visualized using iTOL v7.2 (https://itol.embl.de).

To evaluate the timing of emergence of La Réunion 2024-2025 epidemic clade, we reconstructed time-scaled phylogenies with BEAST (v1.10.5, [10]), using a subset of 173 sequences from La Réunion sampled between the start (August 2024) and the peak of the epidemic (April 2025), and 16 sequences from cases imported from the island into metropolitan France. We first performed a root-to-tip analysis and removed 2 sequences whose sampling date was incongruent with their genetic divergence resulting in a subset of 187 sequences exhibiting sufficient association between genetic distances and sampling dates (correlation=0.57, supplementary Figure S7) to perform bayesian inference. We performed phylogenetic inference under two different substitution models (the HKY substitution model with a gamma-distributed rate variation among sites and no partition into codon positions (HKYG4), or the Shapiro-Rambaut-Drummond-2006 (SRD06)), with either a strict or an uncorrelated lognormal (UCLN) clock model, and used an exponential growth coalescence model. For each model, we ran one MCMC chain of 50 million states with the BEAGLE computational library [11]. We used Tracer (v1.7, [12]) for inspecting convergence and mixing, discarding the first 10 % of steps as burn-in, and ensuring that estimated sampling size (ESS) values associated with estimated parameters were all >200. To identify the best fitted model we performed marginal likelihood estimation using path sampling/ stepping-stone sampling. All xml files for these analyses are available at https://github.com/rklitting/CHIKV_Reunion_2025_RC.

Supplementary tables

ID	Virus	Sampling Territory	Sampling date	Accession number	Sequencing technology
S5b01	CHIKV	REU	2024-11-05	PV685534	Nanopore
S5b06	CHIKV	REU	2024-11-14	PV685535	Nanopore
S5b10	CHIKV	REU	2024-11-22	PV685536	Nanopore
S8b04	CHIKV	REU	2024-11-29	PV685537	Nanopore
S8b05	CHIKV	REU	2024-11-27	PV685538	Nanopore
S8b43	CHIKV	REU	2024-12-19	PV685539	Nanopore
S8b46	CHIKV	REU	2024-12-27	PV685540	Nanopore
S8b47	CHIKV	REU	2024-12-26	PV685541	Nanopore
S8b57	CHIKV	REU	2025-01-03	PV685542	Nanopore
S8b84	CHIKV	REU	2024-12-30	PV685543	Nanopore
S8b91	CHIKV	REU	2024-12-19	PV685544	Nanopore
S8b92	CHIKV	REU	2024-12-30	PV685545	Nanopore
S9b06	CHIKV	REU	2025-01-02	PV685546	Nanopore
S9b52	CHIKV	REU	2025-01-14	PV685547	Nanopore
S9b65	CHIKV	REU	2025-01-24	PV685548	Nanopore
S9b84	CHIKV	REU	2025-01-15	PV685549	Nanopore
S10b05	CHIKV	REU	2025-02-03	PV685550	Nanopore
S10b70	CHIKV	REU	2025-01-20	PV685551	Nanopore

S12b21	CHIKV	REU	2024-12-18	PV685552	Nanopore
S12b53	CHIKV	REU	2024-12-16	PV685553	Nanopore
S12b54	CHIKV	REU	2024-12-13	PV685554	Nanopore
S12b64	CHIKV	REU	2025-01-06	PV685555	Nanopore
S15b22	CHIKV	REU	2025-01-30	PV685556	Nanopore
S15b28	CHIKV	REU	2025-01-28	PV685557	Nanopore
S18b10	CHIKV	REU	2025-02-12	PV685558	Nanopore
S20b56	CHIKV	REU	2025-03-17	PV685559	Nanopore
S25b44	CHIKV	REU	2025-03-22	PV685560	Nanopore
S8b66	CHIKV	REU	2025-01-19	PV685561	Nanopore
S9b40	CHIKV	REU	2025-01-14	PV685562	Nanopore
S10b25	CHIKV	REU	2025-02-05	PV685563	Nanopore
S13b17	CHIKV	REU	2025-02-20	PV685564	Nanopore
S13b68	CHIKV	REU	2025-02-24	PV685565	Nanopore
S18b28	CHIKV	REU	2025-02-10	PV685566	Nanopore
S19b28	CHIKV	REU	2025-02-18	PV685567	Nanopore
S16b66	CHIKV	REU	2025-03-07	PV685568	Nanopore
S9b09	CHIKV	REU	2025-01-28	PV685569	Nanopore
S10b38	CHIKV	REU	2025-02-09	PV685570	Nanopore
S19b29	CHIKV	REU	2025-02-19	PV685571	Nanopore

S10b75	CHIKV	REU	2025-01-20	PV685572	Nanopore
S16b39	CHIKV	REU	2025-03-09	PV685573	Nanopore
S16b41	CHIKV	REU	2025-03-06	PV685574	Nanopore
S19b02	CHIKV	REU	2025-03-12	PV685575	Nanopore
S8b26	CHIKV	REU	2024-12-12	PV685576	Nanopore
S9b24	CHIKV	REU	2024-12-26	PV685577	Nanopore
S12b45	CHIKV	REU	2025-01-27	PV685578	Nanopore
S14b43	CHIKV	REU	2025-03-02	PV685579	Nanopore
S9b61	CHIKV	REU	2025-01-24	PV685580	Nanopore
S9b80	CHIKV	REU	2025-01-22	PV685581	Nanopore
S9b64	CHIKV	REU	2025-01-24	PV685582	Nanopore
S13b84	CHIKV	REU	2025-02-26	PV685583	Nanopore
S19b39	CHIKV	REU	2025-02-19	PV685584	Nanopore
S16b90	CHIKV	REU	2025-02-10	PV685585	Nanopore
S15b91	CHIKV	REU	2025-03-05	PV685586	Nanopore
S22b92	CHIKV	REU	2025-03-19	PV685587	Nanopore
S15b29	CHIKV	REU	2025-01-31	PV685588	Nanopore
S20b67	CHIKV	REU	2025-03-06	PV685589	Nanopore
S25b45	CHIKV	REU	2025-03-22	PV685590	Nanopore
S21b86	CHIKV	REU	2025-02-25	PV685591	Nanopore

S20b04	CHIKV	REU	2025-03-14	PV685592	Nanopore
S14b04	CHIKV	REU	2025-03-01	PV685593	Nanopore
S5b09	CHIKV	REU	2024-11-18	PV685594	Nanopore
S8b01	CHIKV	REU	2024-11-25	PV685595	Nanopore
S8b03	CHIKV	REU	2024-11-23	PV685596	Nanopore
S8b06	CHIKV	REU	2024-11-27	PV685597	Nanopore
S8b10	CHIKV	REU	2024-12-03	PV685598	Nanopore
S8b13	CHIKV	REU	2024-12-04	PV685599	Nanopore
S8b14	CHIKV	REU	2024-12-06	PV685600	Nanopore
S8b15	CHIKV	REU	2024-12-06	PV685601	Nanopore
S8b17	CHIKV	REU	2024-12-07	PV685602	Nanopore
S8b29	CHIKV	REU	2024-12-14	PV685603	Nanopore
S8b31	CHIKV	REU	2024-12-14	PV685604	Nanopore
S8b49	CHIKV	REU	2025-01-02	PV685605	Nanopore
S8b75	CHIKV	REU	2024-12-23	PV685606	Nanopore
S9b04	CHIKV	REU	2025-01-02	PV685607	Nanopore
S9b11	CHIKV	REU	2025-01-04	PV685608	Nanopore
S9b14	CHIKV	REU	2025-01-07	PV685609	Nanopore
S9b21	CHIKV	REU	2024-12-26	PV685610	Nanopore
S9b41	CHIKV	REU	2025-01-13	PV685611	Nanopore

S9b46	CHIKV	REU	2024-12-18	PV685612	Nanopore
S9b78	CHIKV	REU	2025-01-28	PV685613	Nanopore
S10b66	CHIKV	REU	2025-01-13	PV685614	Nanopore
S10b67	CHIKV	REU	2025-01-06	PV685615	Nanopore
S12b09	CHIKV	REU	2025-01-06	PV685616	Nanopore
S12b15	CHIKV	REU	2024-12-18	PV685617	Nanopore
S12b25	CHIKV	REU	2024-12-27	PV685618	Nanopore
S12b39	CHIKV	REU	2024-10-24	PV685619	Nanopore
S12b41	CHIKV	REU	2024-09-26	PV685620	Nanopore
S12b63	CHIKV	REU	2024-12-12	PV685621	Nanopore
S12b65	CHIKV	REU	2024-11-25	PV685622	Nanopore
S12b70	CHIKV	REU	2024-12-04	PV685623	Nanopore
S12b75	CHIKV	REU	2024-12-05	PV685624	Nanopore
S12b78	CHIKV	REU	2024-12-11	PV685625	Nanopore
S14b32	CHIKV	REU	2025-02-26	PV685626	Nanopore
S15b60	CHIKV	REU	2025-02-08	PV685627	Nanopore
S15b61	CHIKV	REU	2025-02-12	PV685628	Nanopore
S17b13	CHIKV	REU	2025-01-31	PV685629	Nanopore
S17b18	CHIKV	REU	2025-02-03	PV685630	Nanopore
S17b75	CHIKV	REU	2025-02-15	PV685631	Nanopore

S18b11	CHIKV	REU	2025-02-12	PV685632	Nanopore
S18b33	CHIKV	REU	2025-02-19	PV685633	Nanopore
S22b46	CHIKV	REU	2025-03-19	PV685634	Nanopore
S8b07	CHIKV	REU	2024-11-30	PV685635	Nanopore
S8b22	CHIKV	REU	2024-12-12	PV685636	Nanopore
S9b50	CHIKV	REU	2024-12-18	PV685637	Nanopore
S8b93	CHIKV	REU	2025-01-03	PV685638	Nanopore
S13b54	CHIKV	REU	2025-02-24	PV685639	Nanopore
S15b64	CHIKV	REU	2025-01-10	PV685640	Nanopore
S9b49	CHIKV	REU	2024-12-18	PV685641	Nanopore
S9b13	CHIKV	REU	2025-01-08	PV685642	Nanopore
S9b19	CHIKV	REU	2024-12-13	PV685643	Nanopore
S9b81	CHIKV	REU	2025-01-15	PV685644	Nanopore
S17b30	CHIKV	REU	2025-03-08	PV685645	Nanopore
S22b42	CHIKV	REU	2025-03-18	PV685646	Nanopore
S10b17	CHIKV	REU	2025-02-04	PV685647	Nanopore
S17b74	CHIKV	REU	2025-02-13	PV685648	Nanopore
S8b89	CHIKV	REU	2024-12-28	PV685649	Nanopore
S9b07	CHIKV	REU	2025-01-08	PV685650	Nanopore
S12b81	CHIKV	REU	2024-12-07	PV685651	Nanopore

S9b79	CHIKV	REU	2025-01-21	PV685652	Nanopore
S19b10	CHIKV	REU	2025-02-18	PV685653	Nanopore
S10b29	CHIKV	REU	2025-02-08	PV685654	Nanopore
S10b74	CHIKV	REU	2025-01-20	PV685655	Nanopore
S21b42	CHIKV	REU	2025-02-25	PV685656	Nanopore
S10b02	CHIKV	REU	2025-01-30	PV685657	Nanopore
S19b50	CHIKV	REU	2025-03-13	PV685658	Nanopore
S8b51	CHIKV	REU	2025-01-06	PV685659	Nanopore
S8b82	CHIKV	REU	2024-12-27	PV685660	Nanopore
S10b30	CHIKV	REU	2025-02-08	PV685661	Nanopore
S23b83	CHIKV	REU	2025-03-20	PV685662	Nanopore
S20b13	CHIKV	REU	2025-03-15	PV685663	Nanopore
S15b86	CHIKV	REU	2025-03-05	PV685664	Nanopore
S20b30	CHIKV	REU	2025-03-15	PV685665	Nanopore
S19b42	CHIKV	REU	2025-03-13	PV685666	Nanopore
S17b24	CHIKV	REU	2025-02-10	PV685667	Nanopore
S8b64	CHIKV	REU	2025-01-13	PV685668	Nanopore
S19b17	CHIKV	REU	2025-02-19	PV685669	Nanopore
S16b60	CHIKV	REU	2025-03-07	PV685670	Nanopore
S27b31	CHIKV	REU	2025-02-17	PV685671	Nanopore

S26b04	CHIKV	REU	2025-04-12	PV685672	Nanopore
S26b44	CHIKV	REU	2025-04-04	PV685673	Nanopore
S27b91	CHIKV	REU	2025-03-13	PV685674	Nanopore
S28b13	CHIKV	REU	2025-03-13	PV685675	Nanopore
S30b73	CHIKV	REU	2025-03-30	PV685676	Nanopore
S28b69	CHIKV	REU	2025-03-24	PV685677	Nanopore
S26b05	CHIKV	REU	2025-04-12	PV685678	Nanopore
S26b07	CHIKV	REU	2025-04-13	PV685679	Nanopore
S26b08	CHIKV	REU	2025-04-13	PV685680	Nanopore
S26b12	CHIKV	REU	2025-04-06	PV685681	Nanopore
S2b92	CHIKV	REU	2024-08-27	PV685682	Nanopore
S2b93	CHIKV	REU	2024-09-03	PV035814	Nanopore
S3b82	CHIKV	REU	2024-09-16	PV685684	Nanopore
S3b83	CHIKV	REU	2024-09-12	PV685685	Nanopore
S4b3	CHIKV	REU	2024-10-25	PV685686	Nanopore
S06b01	CHIKV	REU	2024-08-14	PV685687	Nanopore
S5b07	CHIKV	REU	2024-11-18	PV685688	Nanopore
S5b08	CHIKV	REU	2024-11-16	PV685689	Nanopore
S29b77	CHIKV	REU	2025-03-27	PV685690	Nanopore
S30b86	CHIKV	REU	2025-03-31	PV685691	Nanopore

S30b84	CHIKV	REU	2025-03-30	PV685692	Nanopore
S27b25	CHIKV	REU	2025-03-24	PV685693	Nanopore
S28b63	CHIKV	REU	2025-03-26	PV685694	Nanopore
S26b16	CHIKV	REU	2025-04-02	PV685695	Nanopore
S28b85	CHIKV	REU	2025-03-26	PV685696	Nanopore
S30b93	CHIKV	REU	2025-03-31	PV685697	Nanopore
S26b09	CHIKV	REU	2025-04-06	PV685698	Nanopore
S26b70	CHIKV	REU	2025-03-21	PV685699	Nanopore
S26b18	CHIKV	REU	2025-03-31	PV685700	Nanopore
S28b03	CHIKV	REU	2025-04-09	PV685701	Nanopore
S30b03	CHIKV	REU	2025-04-14	PV685702	Nanopore
S28b02	CHIKV	REU	2025-04-07	PV685703	Nanopore
S28b86	CHIKV	REU	2025-03-25	PV685704	Nanopore
S26b01	CHIKV	REU	2025-04-14	PV685705	Nanopore
S26b15	CHIKV	REU	2025-04-06	PV685706	Nanopore

Supplementary Table S2. Sequence data from La Réunion island. Virus genomes are also available at https://github.com/rklitting/CHIKV_Reunion_2025_RC. Territory ISO code 3 correspondence: REU: Réunion.

Substitution model	Clock	Median	95%HPD	Log marginal likelihood (PS)	Log marginal likelihood (SS)
HKY+G4	Strict	2024-08-07	[2024-07-09;2024-08-14]	-16917.2884653775	-16918.5182256716
SRD06	Strict	2024-08-07	[2024-07-05;2024-08-14]	-16920.4226230075	-16921.6880010353 0
HKY+G4	UCLN	2024-08-07	[2024-07-06;2024-08-14]	-16943.4472816490	-16944.6366898631
SRD06	UCLN	2024-08-03	[2024-07-07;2024-08-14]	-16924.3517289319	-16925.5266307514

Supplementary Table S3: TMRCA estimates from BEAST analyses under different substitution models and coalescent tree priors. Shown for each coalescent tree prior is the median, with the 95% highest probability distribution of TMRCA in parentheses. Also shown is the log marginal likelihood obtained using path-sampling and stepping-stone sampling for each model/prior combination.

ID	Virus	Sampling Territory	Travel location	Sampling date	Accession number	Sequencing technology
79901-1	CHIKV	mainland FRA	REU	2024-11-26	PV700160	Ion Torrent
80698-2	CHIKV	mainland FRA	REU	2025-03-25	PV700167	Ion Torrent
80889-4	CHIKV	mainland FRA	REU	2025-04-06	PV700172	Ion Torrent
80905-1	CHIKV	mainland FRA	REU	2025-04-03	PV700175	Ion Torrent
80652-1	CHIKV	mainland FRA	REU	2025-03-23	PV700165	Ion Torrent
80773-5	CHIKV	mainland FRA	REU	2025-03-28	PV700168	Ion Torrent
80893-2	CHIKV	mainland FRA	REU	2025-04-02	PV700173	Ion Torrent

80801-1	CHIKV	mainland FRA	REU	2025-03-25	PV700169	Ion Torrent
80897-3	CHIKV	mainland FRA	REU	2025-04-05	PV700174	Ion Torrent
80835-5	CHIKV	mainland FRA	REU	2025-04-03	PV700170	Ion Torrent
80618-3	CHIKV	mainland FRA	REU	2025-03-20	PV700164	Ion Torrent
80844-1	CHIKV	mainland FRA	REU	2025-04-01	PV700171	Ion Torrent
80695-2	CHIKV	mainland FRA	REU	2025-03-25	PV700166	Ion Torrent
80278-3	CHIKV	mainland FRA	REU	2025-02-23	PV700161	Ion Torrent
80480-1	CHIKV	mainland FRA	REU	2025-03-13	PV700162	Ion Torrent
80595-1	CHIKV	mainland FRA	REU	2025-03-18	PV700163	Ion Torrent
S27b01	CHIKV	MAY	REU	2025-04-11	PV685522	Nanopore
S27b02	CHIKV	MAY	REU	2025-04-11	PV685523	Nanopore
S27b03	CHIKV	MAY	REU	2025-04-01	PV685524	Nanopore
S27b04	CHIKV	MAY	REU	2025-03-29	PV685525	Nanopore
S27b05	CHIKV	MAY		2025-03-22	PV685526	Nanopore
S27b07	CHIKV	MAY	REU	2025-03-28	PV685527	Nanopore
S27b08	CHIKV	MAY		2025-03-27	PV685528	Nanopore
S27b09	CHIKV	MAY	REU	2025-03-27	PV685529	Nanopore
S27b10	CHIKV	MAY		2025-04-12	PV685530	Nanopore

S27b11	CHIKV	MAY	REU	2025-04-10	PV685531	Nanopore
S27b12	CHIKV	MAY	REU	2025-03-20	PV685532	Nanopore
S27b13	CHIKV	MAY	REU	2025-04-11	PV685533	Nanopore

Supplementary Table S4. Sequence data from other territories including Mayotte and Mainland France. Virus genomes are also available at https://github.com/rklitting/CHIKV_Reunion_2025_RC. Territory ISO code 3 correspondence: FRA: France, MAY: Mayotte, REU: Réunion. For individuals with a history of travel to La Réunion within a time frame compatible with an infection abroad, if they were residing in mainland France, the source of the infection was considered to be La Réunion.

Primer couple	Name	Sequence
Couple 1	GP-CHIKV-1S	GCAAAGCAAGAGATTAAGAACC
	GP-CHIKV-1458R	CTAGTCCTCAAAGGGATTGAC
Couple 2	GP-CHIKV-1251S	TAAGTGGGCTAAGGAGTGCC
	GP-CHIKV-2966R	GTCTTTATCCACGGGTCGCC
Couple 3	GP-CHIKV-2915S	GTACTCCTAACACGAACGGAAG
	GP-CHIKV-4540R	CTTGGGTCCGCATCTGTATG
Couple 4	GP-CHIKV-4481S	GATGCAGACGTGGTCATCTAC
	GP-CHIKV-6016R	GGACAATCGAACGTTGATCGG
Couple 5	GP-CHIKV-5919S	GACTATACTTAATGTCAGAGACC
	GP-CHIKV-7543R	GTAGCTGATTAGTGTTTAGGTAC
Couple 6	GP-CHIKV-7486S	CGGCGGTCCTAAATAGGTAC
	GP-CHIKV-8987R	CGTGCTGCAAGGTAACTCTC
Couple 7	GP-CHIKV-8805S	AGCACCGTGCACGATTACTG
	GP-CHIKV-9993R	GATCACTGTTACGTGTTCGTAC
Couple 8	GP-CHIKV-9901S	GTCCTATGCAACTGTCTGAGAC
	GP-CHIKV-11788R	ATCTCCTACGTCCCTGTGGG

Supplementary Table S5. Amplification primers for whole genome sequencing of CHIKV in the Arbovirus NRC – mainland France.

Pool	Name	Sequence
Pool A	CHIKV_1_LEFT_1	CTACCAGTTTCTTACTGCTCTACTCT
Pool A	CHIKV_1_RIGHT_1	CCTCTTGTAGACCGTGTGTGT
Pool B	CHIKV_2_LEFT_1	CCTTCAGTAAGTGGGCAAAGGA
Pool B	CHIKV_2_RIGHT_1	ACTTTCTGCCTTGGTCTCACCA
Pool A	CHIKV_3_LEFT_1	GAGATATCTGCACGTACGGTTGA
Pool A	CHIKV_3_RIGHT_1	CGCTACCCAGGTGACTCTCTTA
Pool B	CHIKV_4_LEFT_1	ATACCGGCCAACAGGAGACTAC

Pool B	CHIKV_4_RIGHT_1	CCTCTGTTTGCTTTGGCCACA
Pool A	CHIKV_5_LEFT_1	TGGCAGGCAGAAAAGGATACAG
Pool A	CHIKV_5_RIGHT_1	TACCTGCTTCTGTTGGCCATG
Pool B	CHIKV_6_LEFT_1	CGGTAAACACCCTGGAGGAAGT
Pool B	CHIKV_6_RIGHT_1	TTCATCATGGCGCCGAACTT
Pool A	CHIKV_7_LEFT_1	AGCCTCCTTTGATAAGAGCCAAG
Pool A	CHIKV_7_RIGHT_1	ACTTCATGTGCACGGGTATCTG
Pool B	CHIKV_8_LEFT_1	TGGTGGGGGACAAAGTAATGAA
Pool B	CHIKV_8_RIGHT_1	TGCAGTTATTGATCACTTTGTCTGT
Pool A	CHIKV_9_LEFT_1	AGATAGAGGTACACATGCCCCC
Pool A	CHIKV_9_RIGHT_1	ACATGTGCTTCGCTCAATTGC
Pool B	CHIKV_10_LEFT_1	GAGCGCGTACGAACACGTAA
Pool B	CHIKV_10_RIGHT_1	TTAGTGCCTGCTRAACGACACG
Pool A	CHIKV_11_LEFT_1	TGCAGAAGATTACGGGAGGAGT
Pool A	CHIKV_11_RIGHT_1	TTTTTTTTTTTTTTTTTTTTTTTTTGAAATATTAAAAACAAAAT

Supplementary Table S6. Amplification primers for whole genome sequencing of CHIKV in the Arbovirus NRC - Réunion.

Supplementary figure

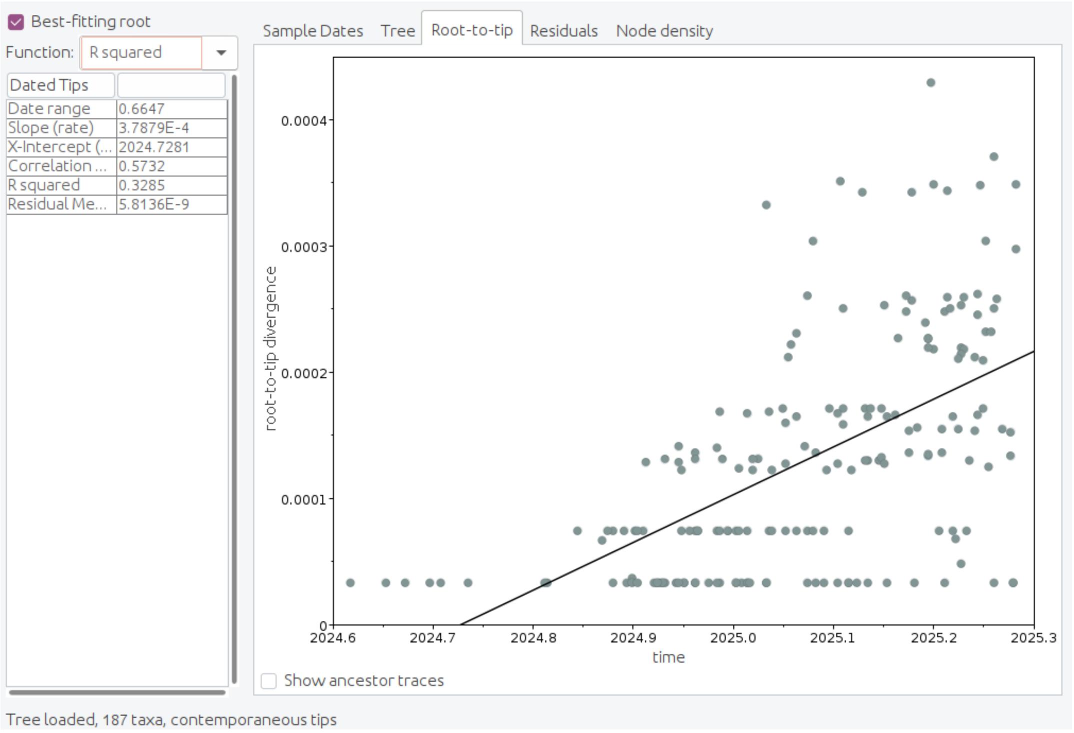

Supplementary Figure S7. Root-to-tip analysis of sequences used for bayesian inference. Regression of genetic distance against time for all sequences included in the dataset used for bayesian inference.

References

1. Johannes Köster, Sven Rahmann, Snakemake—a scalable bioinformatics workflow engine, *Bioinformatics*, Volume 28, Issue 19, October 2012, Pages 2520–2522, https://doi.org/10.1093/bioinformatics/bts480

2. arXiv:1303.3997

3. Grubaugh, N.D., Gangavarapu, K., Quick, J. et al. An amplicon-based sequencing framework for accurately measuring intrahost virus diversity using PrimalSeq and iVar. Genome Biol 20, 8 (2019). https://doi.org/10.1186/s13059-018-1618-7

4. Katoh K, Standley DM. MAFFT multiple sequence alignment software version 7: improvements in performance and usability. Mol Biol Evol. 2013 Apr;30(4):772-80. doi: 10.1093/molbev/mst010. Epub 2013 Jan 16.

5. Larsson, A. (2014). AliView: a fast and lightweight alignment viewer and editor for large data sets. *Bioinformatics*30(22): 3276-3278. http://dx.doi.org/10.1093/bioinformatics/btu531

6. Chernomor O, Minh BQ, Forest F, Klaere S, Ingram T, Henzinger M, and von Haeseler A (2015) Split diversity in constrained conservation prioritization using integer linear programming. *Methods Ecol. Evol.*, 6, 83-91. DOI: 10.1111/2041-210X.12299

7-8. Nguyen LT, Schmidt HA, von Haeseler A, Minh BQ, IQ-TREE: A Fast and Effective Stochastic Algorithm for Estimating Maximum-Likelihood Phylogenies, Molecular Biology and Evolution, Volume 32, Issue 1, January 2015, Pages 268–274

9. Minh BQ, Schmidt HA, Chernomor O, Schrempf D, Woodhams MD, von Haeseler A, et al. IQ-TREE 2: New Models and Efficient Methods for Phylogenetic Inference in the Genomic Era, Molecular Biology and Evolution, Volume 37, Issue 5, May 2020, Pages 1530–1534

10. Suchard MA, Lemey P, Baele G, et al.. Bayesian phylogenetic and phylodynamic data integration using BEAST 1.10. Virus Evol. 2018;4:vey016), doi: 10.1093/ve/vey016

11. Ayres DL, Darling A, Zwickl DJ, et al.. BEAGLE: an application programming interface and high-performance computing library for statistical phylogenetics. Syst Biol. 2012;61:170–173. doi: 10.1093/sysbio/syr100

12. Rambaut A, Drummond AJ, Xie D, Baele G and Suchard MA (2018) Posterior summarisation in Bayesian phylogenetics using Tracer 1.7. *Systematic Biology*. Syy032
